# Book Reviews

**Published:** 1915-07

**Authors:** 


### BOOK REVIEWS

THE CANCER PROBLEM. By William Seaman Bainbridge, A. M., Sc.D., M. D., Professor of Surgery, New York Polytechnic Medical School and Hospital; Surgeon and Secretary of Committee of Scientific Research, New York Skin and Cancer Hospital; Consulting Surgeon, Manhattan State Hospital, Ward's Island; Honorary President, First International Congress for the Study of Tumors and Cancers, Heidleberg, 1906. 8vo volume of 534 pages and 38 plates. New York. The MacMillan Co. 1914. Cloth, $4.00 net.

Almost any review of this book would be to damn it with faint praise. The Author has worked hard and studied deeply and knows how to write. He has placed before us without any waste of words a comprehensive study of the Cancer Problem as it is to-day. Just a casual glance at the Bibliography of 58 closely printed pages makes one gasp and determine to go carefully over the body of the book. The History and the Statistical Consideration chapters are illuminating and furnish material with which the physician should be able to begin the education of his patients and lay friends in this important matter. The idea of the book seems to be to teach all that is known of cancer and then to lead men who have been accepting its existence as inevitable to do better things and join in a campaign for its eradication. It is true that not much is known of the cause, but the fact that the best minds in the country have centered their efforts on this problem means that the way is clearing for a complete discovery of the disease. The hopeful note is sounded all through the book and one feels the enthusiasm of the author stimulating him to renewed efforts and more careful work as he reads. It is a book for every physician and he need never to be a surgeon or know how to hold a scalpel, clamp a haemostat or judge a paste to make this of value to him. Its educational value among one's patients makes it of universal attractiveness. It shows how to set aside and sweep away the mistaken ideas as to cancer and with the clear field thus opened the work can go forward unhampered.

The book should be read, not reviewed, and no quotations are made on that account. We haven't space to print the whole thing.

---

A MANUAL OF DISEASES OF INFANTS AND CHILDREN. By John Ruhrah, M. D., Professor of Diseases of Children, College of Physicians and Surgeons, Baltimore, Md. Fourth Edition, Thoroughly Revised. 12mo volume of 552 pages, 175 illustrations. Philadelphia and London. W. B. Saunders Company, 1915. Cloth, $2.50 net.

The several editions of this book during the last ten years show its popularity and usefulness. In this the fourth edition, the work has been brought entirely up to date and one has in compact and handy form a reliable epiiome of the subject of Diseases of Children.

As is stated in the Preface, the medical student has so many things to think about and to learn that a full treatise with exhaustive essays upon every topic is appalling to him and he does not know what to take and what to leave. This book selects for him and after stating each case clearly and exactly, it still offers to him the opportunity through its references, to make as extended a study as his needs or taste may require. To the practioner, it presents briefly and clearly the subjects it takes up and while speaking authoritatively of the pediatrics of the day, it suggests and recalls to his mind all that he may have read on the subject before.

Its convenient size and weight make it a book for the desk rather than the case and its subject matters will justify its close relation to the daily work.

OUTLINES OF INTERNAL MEDICINE FOR THE USE OF NURSES. By Clifford Bailey Farr, A. M., M. D., Instructor in Medicine, University of Pennsylvania; Assistant Visiting Physician, Philadelphia General Hospital; Pathologist to the Presbyterian Hospital, etc. 12mo volue of 408 pages illustrated with 71 engravings and 5 plates. Philadelphia and New York, Lea & Febiger, 1915. Cloth.

This book is one of the Nurses Text Book Series and covers the ground so well that it is worth the practitioners while to study it. It seems to take for granted that the nurses should have a good preliminary education so that it can convey its message to intelligent people in a brief and succinct manner. Thus it comprises an unusual amount of information in a small space and constitutes itself a valuable guide to any nurse whether she is undergraduate and studying or established and in need of reliable reference.

Its system of "Parts" is a pleasing one and keeps the student in mind of exactly what she is doing. Eight Parts have to do with diseases of the various internal systems of the body and two concern themselves with harmful agencies invading the body from without. Thus from the beginning the reader is led to think of invasions that should be avoided and dangers that must be guarded against and all idea of kismet is set aside. Often enough nurses go to training with the household ideas of disease so deeply settled in their minds that unless a definite teaching to the contrary is given they graduate with the belief in the heredity of tuberculosis or in the necessity for children to have the exanthemata. This "without" classification is a splendid one and we welcome its presentation in this book.

